# Shining light on the noradrenergic system

**DOI:** 10.1117/1.NPh.10.4.044406

**Published:** 2023-09-26

**Authors:** Emmeraude Tanguay, Sarah-Julie Bouchard, Martin Lévesque, Paul De Koninck, Vincent Breton-Provencher

**Affiliations:** a CERVO Brain Research Centre, Quebec, Quebec, Canada; b Université Laval, Department of Psychiatry and Neuroscience, Faculty of Medicine, Quebec, Quebec, Canada; c Université Laval, Department of Biochemistry, Microbiology, and Bioinformatics, Faculty of Science and Engineering, Quebec, Quebec, Canada

**Keywords:** noradrenaline, norepinephrine, locus coeruleus, calcium imaging, genetically encoded noradrenaline sensors, two-photon microscopy, fiber photometry, behavior, sleep, arousal, stress, learning, memory

## Abstract

Despite decades of research on the noradrenergic system, our understanding of its impact on brain function and behavior remains incomplete. Traditional recording techniques are challenging to implement for investigating *in vivo* noradrenergic activity, due to the relatively small size and the position in the brain of the locus coeruleus (LC), the primary location for noradrenergic neurons. However, recent advances in optical and fluorescent methods have enabled researchers to study the LC more effectively. Use of genetically encoded calcium indicators to image the activity of noradrenergic neurons and biosensors that monitor noradrenaline release with fluorescence can be an indispensable tool for studying noradrenergic activity. In this review, we examine how these methods are being applied to record the noradrenergic system in the rodent brain during behavior.

## 1 Introduction

The forebrain noradrenaline (NA) system primarily originates from neurons located in the locus coeruleus (LC). LC neurons produce a diverse range of projections that result in NA innervation of numerous cortical and subcortical areas.^1^^–^^3^ Despite the extensive projection network, the conditions under which NA is released and the corresponding behavioral contexts have been difficult to characterize. Studies using perturbation techniques and electrophysiological recordings of LC neurons have suggested that LC is involved in innate behaviors such as sleep,^4^^–^^10^ arousal,^6^^,^^11^^–^^14^ stress^15^^–^^19^ and feeding,^20^^,^^21^ as well as cognitive processes including attention,^22^^–^^25^ learning,^26^^–^^30^ and memory.^27^^,^^30^^–^^32^ To refine our understanding of the function of the NA system, it is critical to develop novel recording techniques that can accurately and reliably monitor the activity of identified LC-NA neurons *in vivo*.

**Fig. 1:**
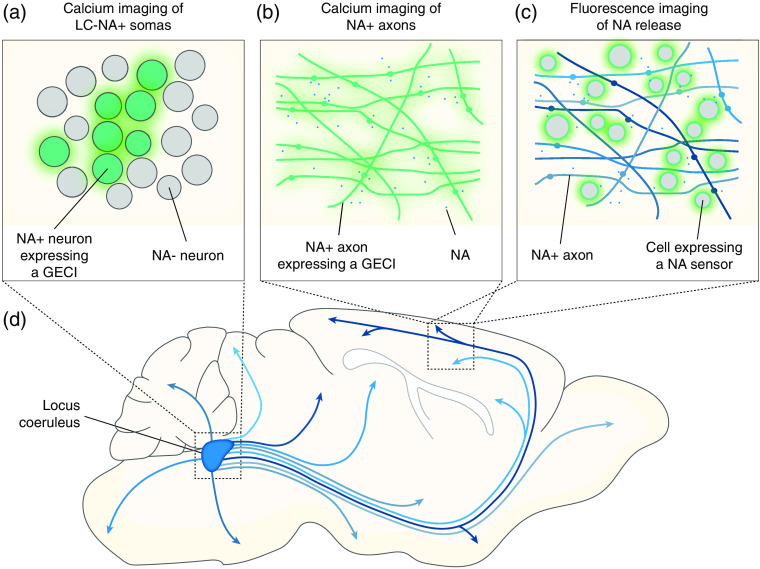
Monitoring noradrenaline (NA) with light. Various techniques to monitor the NA system. (a) LC somatic activity imaged with a genetically encoded calcium indicator (GECI). (b) Imaging of NA+ axons expressing a GECI. (c) Imaging NA release in target regions with G-protein coupled receptor (GPCR)-based biosensors. (d) Illustration of the LC projection system.

The LC has a width of only 300  μm in mice^33^ and 1 mm in humans,^34^ and is located deep in the pons, making it challenging to target with electrodes using stereotaxic coordinates. In addition, LC-NA neurons are intermingled with neurons expressing gamma-aminobutyric acid (GABA)^12^^,^^35^^–^^37^ and other types of neurons,^38^^–^^40^ which can contaminate extracellular single-unit recordings with non-NA releasing neurons. While photo-tagging, a method that combines electrophysiology and optogenetics to record from genetically identified neuronal populations,^41^^,^^42^ has been used to record from LC-NA neurons, it only yields a limited number of identified neurons per recordings.^12^^,^^26^^,^^28^^,^^32^^,^^43^^,^^44^ Therefore, neurophotonics has democratized research on the LC, making it more accessible to researchers beyond a few specialized labs. In this review, we will discuss two methods that have been applied to record LC-NA activity in the rodent brain and how they have advanced LC research. First, we will discuss how recent research has used genetically encoded calcium indicators (GECIs) to monitor the activity of LC-NA neurons and their projections with various imaging methods [Figs. 1(a) and 1(b)]. Second, we will discuss the development of NA biosensors and how they have been applied to LC research [Fig. 1(c)].

## 2 Illuminating LC Neuron Activity

GECIs are widely used to visualize neuronal activity, including LC-NA neurons.^45^^,^^46^ By genetically targeting these indicators to NA cells, researchers can monitor their activity during behavior. Various mouse lines have been used to genetically access LC-NA neurons through virus injections, such as the dopamine beta-hydoxylase (DBH)-Cre mouse line where the Cre recombinase is expressed from the dopamine beta hydroxylase locus,^47^^,^^48^ and the norepinephrine transporter (NET)-Cre mouse line that uses the NA transporter locus.^22^^,^^49^ Although the tyrosine hydroxylase (TH)-Cre lines,^47^^,^^50^ where Cre is expressed from the tyrosine hydroxylase locus, have also been used, recent evidence indicates lower specificity in targeting LC-NA neurons using this approach.^51^ As an alternative to mouse lines expressing Cre recombinase, the synthetic DBH promoter PRSx8^52^ could be used to efficiently target LC-NA neurons,^7^^,^^51^^,^^53^ but it has not yet been tested for expressing calcium indicators.

Once a calcium indicator is introduced into LC-NA neurons, calcium dynamics can be assessed using either fiber photometry,^4^^,^^20^^,^^54^^–^^56^ providing population-level activity of LC-NA neurons, or through microendoscopy, providing spatially resolved signals from each LC-NA neuron.^26^^,^^57^ These measurements conducted at the population level of the LC have allowed researchers to determine the behavioral context in which the NA system is broadly active. Therefore, these techniques have advanced our understanding of LC-NA function in innate behavior such as feeding,^20^ the link between sleep and stress,^4^ and maternal behavior,^54^ as well as LC-NA role in cognitive processes such as sensory plasticity,^55^ learned behavior,^26^ exploitation of a behavior,^57^ and fear memory formation.^56^

One important consideration when measuring the activity of all NA neurons at the level of the LC is that it fails to account for the outputs of the NA system or subcellular differences within LC-NA neurons. Recent anatomical evidence indicates that some LC-NA neurons selectively project to specific regions of the brain.^3^^,^^15^^,^^26^^,^^30^^,^^53^^,^^58^^–^^62^ Furthermore, the activity of LC neurons is not fully correlated between neurons,^30^^,^^43^^,^^63^ and this heterogeneous activity potentially supports functional modularity at the output level.^15^^,^^26^^,^^30^^,^^59^ Therefore, the overall activity of the LC might not be a good predictor for NA release of a specific brain area.

To investigate projection specific activity of the NA system, researchers have quantified calcium activity in axonal projections.^64^ To target LC-NA+ neurons, a strategy similar to somatic calcium imaging can be used, but with extra consideration for the type of calcium indicator. To successfully label LC-NA projections, green fluorescent protein (GFP)-based genetically encoded calcium indicators (GCaMP) that are axon-targeted^65^^,^^66^ or that have a brighter baseline fluorescence (e.g., GCaMP7b)^26^^,^^67^ are preferred. Axonal labeling with GCaMP can be achieved using one of the aforementioned Cre-recombinase mouse lines, but labeling specificity can be improved by injecting a retrograde virus expressing Cre or Flpo in a target area.^68^^–^^70^ Imaging of LC-NA axons expressing GCaMP has been accomplished in the cerebral cortex and the cerebellum using multiphoton imaging through a cranial window, to correlate LC-NA signals with general behavioral states such as arousal and locomotion,^12^^,^^71^^–^^77^ with sensorimotor learning^26^^,^^66^ and with spatial reward learning.^27^ In addition, fiber photometry has been used in freely moving animals to image LC-NA projections to the hippocampus during memory formation.^56^

In addition to LC axonal imaging, it is possible to record activity from selected populations of LC-NA neurons using a microendoscope implanted at the surface of the LC.^26^^,^^57^ This approach would allow for a comparison of the activity of projection-specific LC neurons within the same animal. While this method is feasible in practice, to date, we have not observed any labs applying microendoscopy in this context.

## 3 Monitoring the Release of Noradrenaline with Light

Electrophysiological recordings and the imaging of GECIs are instrumental for determining the link between behavior and LC-NA activity. However, one important question remains as to what the underlying dynamics of NA release associated with this activity are. Indeed, the cellular mechanisms governing neurotransmitter release are complex, and the release of NA could be not fully proportional to the firing activity of LC-NA neurons. This has been observed for the dopaminergic system where cellular mechanisms present in axons can affect dopamine release.^78^^,^^79^ Therefore, methods that directly assess the release of neurotransmitters are critical for understanding NA dynamics. The use of classic detection methods, such as microdialysis-coupled biochemical analysis, has allowed the study of NA release in target areas,^80^^–^^82^ but the poor temporal and spatial resolution has prevented our understanding of the fast kinetics of NA release or cellular-level NA signals that occur during behavior. To overcome these limitations, fluorescent biosensors that track extracellular NA dynamics have been developed.

**Fig. 2:**
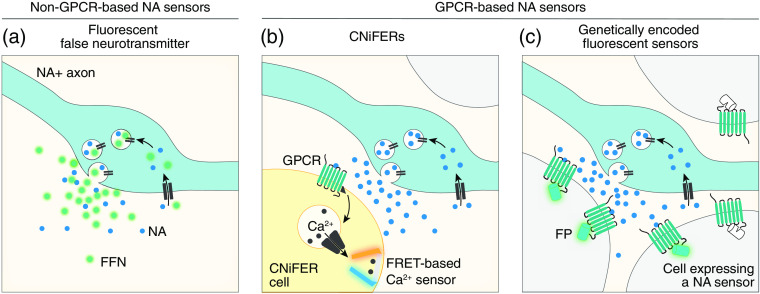
Imaging NA release *in vivo* with light. (a) Imaging NA release from bouton using FFN, a fluorescent substrate for the NA transporter NET and the vesicular monoamine transporter 2. (b) Imaging NA release using a CNiFERs. CNiFER cells expressing a NA GPCR are injected in a target region. Upon binding with NA, the GPCR stimulates the release of calcium inside the cell, which is detected by a FRET-based calcium sensor. (c) Imaging NA release with genetically encoded fluorescent sensors expressed in cells of a target region. Upon binding with NA, the modified GPCR coupled with a fluorescent protein exhibits a large fluorescent increase.

Two types of fluorescent biosensors exist: G-protein coupled receptor (GPCR) and non-GPCR based sensors (Fig. 2). Currently, non-GPCR fluorescent sensors are either made from neurotransmitter nanosensors^83^^,^^84^ or made from false neurotransmitters.^85^^,^^86^ Neurotransmitter nanosensors, which are functionalized carbon nanotubes, have proven effective for detecting dopamine or NA release in cultured neurons^83^ and striatal slices.^84^ However, their lack of selectivity for NA over dopamine poses a challenge when applied to regions containing both neurotransmitters. Moreover, using these nanosensors in the intact brain has not been done yet. On the other hand, fluorescent false neurotransmitter (FFN) are molecules that combines structural features of a neurotransmitter with the fluorescent core of a fluorophore, thus they act as a substrate for neurotransmitter transporters allowing them to enter synaptic vesicles [Fig. 2(a)].^85^^,^^86^ The advantage of FFNs is that they act as a substrate for neurotransmitter transporters allowing them to enter synaptic vesicles, thus they enable the imaging of neurotransmitter dynamics from single release sites. For example, false neurotransmitters enable the imaging of NA dynamics from single axons in anesthetized mice after a systemic injection of amphetamines.^85^ Nonetheless, the use of these methods in awake behaving animals will require further development.

GPCR-based biosensors are a predominant approach for monitoring volume signaling of neurotransmitter release in the brain of awake behaving mice. The first iteration of such a tool in cultured cells used fluorescence resonance energy transfer (FRET) to monitor the conformational switch of alpha-2 receptor when bound to NA.^87^ Application of this concept was then made possible *in vivo* using a cell-based neurotransmitter fluorescent engineered reporters (CNiFERs).^76^^,^^88^^,^^89^ In this approach, cells that express a specific GPCR receptor for the chosen target (NA α1a receptor) trigger an increase in intracellular calcium concentration, which is then detected by a genetically encoded FRET-based ${\mathrm{Ca}}^{2+}$ sensor^88^^,^^89^ [Fig. 2(b)]. These CNiFERs cells can then be implanted in the brain region of interest to quantify the surrounding NA release.^88^^,^^89^ This technique presents a level of specificity and a temporal resolution that allowed previous work to link NA release to LC axonal activity in the cortex.^76^ However, the need to implant exogenous cells in specific brain regions limits the utility of this approach, notably it cannot be combined with local measurements of neuronal activity.

To overcome these limitations, genetically encoded fluorescent sensors have rapidly become a popular set of tools for quantifying neurotransmitter release^90^^–^^92^ [Fig. 2(c)]. Three families of these new sensors exist for monitoring NA—${\mathrm{GRAB}}_{\mathrm{NE}}$,^93^^,^^94^ nLight,^75^^,^^95^^,^^96^ and ${\mathrm{MTRIA}}_{\mathrm{NE}}$^91^—which are modified versions of alpha-1 (nLightG/R), alpha-2 (${\mathrm{GRAB}}_{\mathrm{NE}}$), and beta-2 (nLight and ${\mathrm{MTRIA}}_{\mathrm{NE}}$) adrenergic receptors. These sensors can be stably expressed in specific cell types of the brain for several months, making them compatible with a range of imaging methods, including fiber photometry, two-photon imaging, and widefield imaging. Using either fiber photometry or two-photon imaging, researchers have used these sensors to uncover the temporal dynamics of NA release associated with various behavioral states, such as sleep,^4^^,^^8^^,^^9^ the default mode network,^97^ arousal,^73^^,^^98^ and the processing of aversive stimuli.^75^ These sensors have also been instrumental in demonstrating the link between NA temporal dynamics and learning,^26^^,^^99^ as well as NA and memory consolidation.^8^^,^^100^

By imaging NA sensors in combination with optogenetics, researchers have begun to reveal the link between LC neuronal activity and NA release in target regions.^22^^,^^93^^,^^94^^,^^96^^,^^101^ When combining these tools, it is critical to select optically compatible molecules, to avoid any interference between the excitation wavelengths of the opsin and the sensor. For example, by infecting LC-NA neurons with a red-shifted opsin and expressing ${\mathrm{GRAB}}_{\mathrm{NE}}$ in the thalamus and the basal forebrain, researchers have demonstrated the interaction between the tonic and phasic modes of LC firing and NA release during acute stress exposure.^101^ Multiplexing these biosensors with other optical tools will potentially be transformative for our understanding of the NA system.

Anatomical and functional evidence suggest that NA release is modular, making it promising to measure cortex-wide dynamics of NA release using widefield microscopy of genetically encoded fluorescent sensors.^102^ A similar approach has been implemented for studying the coordination of acetylcholine release and neuronal activity in different behavioral states,^103^ suggesting that widefield microscopy can be used for imaging NA release. A transgenic line expressing the next-generation noradrenaline sensors was recently developed allowing mesoscopic NA and calcium dynamics in dorsal cortex of awake mice.^94^ In addition, multi-site fiber photometry^104^^,^^105^ could be used to track the release of NA in specific brain regions, as it has recently been used for showing visual cortex specific NA signals.^73^ Another important application is the cell-specific expression of NA sensors, which will enable us to determine if the endogenous release of NA differentially affects particular cell types in the brain, such as cortical astrocytes.^73^^,^^75^^,^^77^^,^^98^^,^^106^ Overall, these genetically encoded fluorescent sensors are a powerful tool for investigating NA release dynamics and have the potential to greatly enhance our understanding of the NA system.

## 4 Conclusion and Future Directions

Neurophotonic methods have become an essential asset for studying NA and neurotransmitter systems during behavior. Using GECI, neurophotonics enable targeted recordings of LC-NA neurons and axons, or monitoring fast temporal dynamics of NA release through fluorescent biosensors. As other brain areas, such as nuclei A1, A2, A5, A7, and subcoeruleus, also express NA,^107^^–^^111^ we see great opportunity for discovery by applying similar methods to these subdivisions of the central NA system. On the other hand, with the expansion of the color palette of genetically encoded biosensors, such as non-green GECIs,^112^^,^^113^ red-shifted dopamine and NA sensors,^96^^,^^114^^,^^115^ and far-red genetically encoded voltage indicators,^116^ we expect a multiplication of studies that multiplex neurophotonics methods to measure NA release in conjunction with other brain signals.^98^^,^^101^ Furthermore, the use of genetically encoded fluorescent sensors for NA eliminates the need for transgenic approaches, thus measurements of fast NA dynamics can be performed in any animal models. In summary, neurophotonics methods, in combination with genetically encoded biosensors, have become indispensable for studying the LC-NA system’s function during behavior. As these methods continue to evolve, they hold the potential to provide deeper insights into the underlying mechanisms of disorders associated with NA dysregulation.
